# Green‐Light‐Induced Inactivation of Receptor Signaling Using Cobalamin‐Binding Domains

**DOI:** 10.1002/anie.201611998

**Published:** 2017-03-20

**Authors:** Stephanie Kainrath, Manuela Stadler, Eva Reichhart, Martin Distel, Harald Janovjak

**Affiliations:** ^1^ Synthetic PhysiologyInstitute of Science and Technology Austria (IST Austria)Am Campus 13400KlosterneuburgAustria; ^2^ Innovative Cancer ModelsChildren's Cancer Research Institute (CCRI)Zimmermannplatz 101090ViennaAustria

**Keywords:** cobalamins, optogenetics, photochromism, receptors

## Abstract

Optogenetics and photopharmacology provide spatiotemporally precise control over protein interactions and protein function in cells and animals. Optogenetic methods that are sensitive to green light and can be used to break protein complexes are not broadly available but would enable multichromatic experiments with previously inaccessible biological targets. Herein, we repurposed cobalamin (vitamin B12) binding domains of bacterial CarH transcription factors for green‐light‐induced receptor dissociation. In cultured cells, we observed oligomerization‐induced cell signaling for the fibroblast growth factor receptor 1 fused to cobalamin‐binding domains in the dark that was rapidly eliminated upon illumination. In zebrafish embryos expressing fusion receptors, green light endowed control over aberrant fibroblast growth factor signaling during development. Green‐light‐induced domain dissociation and light‐inactivated receptors will critically expand the optogenetic toolbox for control of biological processes.

Optogenetic methods provide spatially and temporally precise control over molecular processes, cellular signals, and animal behavior by making use of microbial or plant photoreceptor domains that are capable of light‐controlled inter‐ or intramolecular interactions. The currently available domain repertoire allows for blue‐ or red‐light‐induced formation of protein complexes;^1^ light oxygen voltage (LOV) sensing domains and phytochromes (PHYs) homodimerize,^2^ PHYs and cryptochromes (CRYs) heterodimerize with accessory proteins,^3^ and CRYs also oligomerize.^4^ Furthermore, blue‐ or red‐light‐induced dissociation of stable complexes that spontaneously form in the dark has been observed in LOV domains in vitro^5^ and for PHYs and CRYs in yeast screens,^6^ and has been implemented as optogenetic tools using LOV domains and engineered fluorescent proteins.^7^ However, none of the currently used photoreceptor domains are maximally responsive to green light, which constitutes a notorious “blind spot” in optogenetic experiments (Figure 1 a). Herein, we employ cobalamin (vitamin B12) binding domains (CBDs) to mediate receptor interaction in the dark that can be abolished by green light, and apply these domains to regulate signaling pathways in human cells and development in zebrafish embryos.

**Figure 1:**
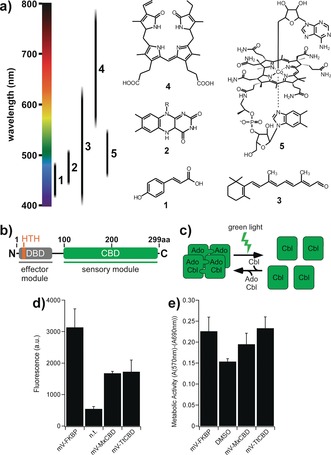
a) Chromophores of main photoreceptor classes (1: *p*‐coumaric acid, 2: flavins, 3: retinal, 4: tetrapyrroles, 5: AdoCbl). b) Domain structure of CarH with light‐sensitive CBD and DNA binding domain (DBD; HTH=helix‐turn‐helix motif), drawn to scale for CarH of *M. xanthus*. c) In the dark, CBDs with bound AdoCbl assemble into tetramers. Upon illumination, the 5′‐deoxyadenosyl group is cleaved, and the complex dissociates. d) Fluorescence intensity of mV‐tagged MxCBD, TtCBD, or FKBP expressed in HEK293 cells, with non‐transfected (n.t.) cells as negative control. e) Metabolic activity of HEK293 cells expressing mV‐MxCBD, mV‐TtCBD, or mV‐FKBP and a 5 % DMSO treated control with reduced viability. Mean values ± SEM for three independent experiments each performed in triplicate are given in (d) and (e).

Functional, photochemical, and structural information on the CBDs of CarH of *Myxococcus xanthus* and *Thermus thermophilus* and the related LitR of *Streptomyces coelicolor* and *Bacillus megaterium* has emerged in recent years, and these proteins represent an active area of photobiology research (Figure 1 b, c).^8^ These transcription factors act as light‐sensitive repressors of carotenoid synthesis. Their relatively small CBDs (ca. 200 amino acids in length) mediate the assembly of dimers of dimers in the dark; these dissociate into monomers upon green‐light irradiation after photolytic cleavage of the 5′‐deoxyadenosylcobalamin (AdoCbl) cofactor (Figure 1 c; see the Supporting Information, Figure S1).8f AdoCbl is an active form of cobalamin that is also synthesized in the mitochondria of eukaryotic cells.^9^ In mammals, cobalamins are required for red blood cell formation, neural function, as well as protein and DNA synthesis, and no adverse effects have been associated with excess cobalamin intake in healthy individuals.^10^ These properties prompted us to explore CBDs in mammalian cells for the green‐light‐induced disruption of signaling processes that were induced in the dark.

We first confirmed that cobalamin supplementation is well tolerated in human embryonic kidney 293 (HEK293) cells. After incubation with AdoCbl or cyanocobalamin (CNCbl, a widely manufactured precursor) at a final concentration of 10 μm for 24 h, we did not observe reduced cell viability (Figure S2; 10 μm corresponds to a typical concentration for cofactor supplementation2b, 3a). We next tested whether CBDs can be expressed robustly and without cytotoxicity by fusing synthetic gene fragments encoding the 618 bp long CBDs of *M. xanthus* (MxCBD) and *T. thermophilus* (TtCBD), which had been codon‐optimized for expression in human cells,2b to the bright yellow fluorescent protein mVenus (mV) to quantify domain expression.^11^ CBD levels approached that of the robustly expressing human FK506 binding protein (FKBP) domain (Figure 1 d) with no measurable cytotoxicity or protein aggregation (Figure 1 e and Figure S3).

We next studied the light‐induced dissociation of a CBD fusion protein complex. Complex formation upon ligand binding is the functional mechanism underlying receptor tyrosine kinase (RTK) activation, leading to events such as mitogen‐activated protein kinase/extracellular signal regulated kinase (MAPK/ERK) signaling.^12^ To control RTK interactions, we fused MxCBD and TtCBD to the C‐terminus (where chemical oligomerization domains or fluorescent proteins had been incorporated beforehand without perturbation of the receptor function) of the intracellular domain (ICD) of the murine fibroblast growth factor receptor 1 (mFGFR1, a prototypical RTK; Figure 2 a).^13^ We replaced the N‐terminal ligand binding and transmembrane domains by a myristoylation (MYR) anchor^11^, 13b so that the receptors are solely controlled by the CBDs. In addition, a C‐terminal hemagglutinin (HA) epitope was incorporated. Interestingly, we found that the fusion proteins were expressed more efficiently in HEK293 cells supplemented with AdoCbl than in cells supplemented with CNCbl or without supplement (Figure 2 b), suggesting that the cofactor facilitates protein expression. This was not observed for mV fusion proteins, likely reflecting a difference in expression yield and stability of the fluorescent protein and receptor ICD fusions (compare Figure S4 and Figure 1 d for experiments with fluorescent protein performed with and without supplementation, respectively).

**Figure 2:**
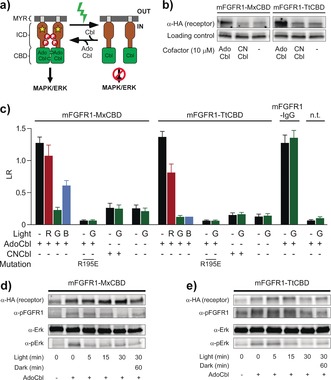
a) CBDs were fused to the ICD of mFGFR1 to engineer receptors that are inactivated by green light. b) Expression of mFGFR1‐MxCBD and mFGFR1‐TtCBD in HEK293 cells supplemented with AdoCbl or CNCbl. c) Activation of the MAPK/ERK pathway (LR=luminescence ratio) by mFGFR1 fused to CBDs or the Fc domain of IgG1 (IgG) and non‐transfected (n.t.) cells in response to red (R; *λ*=670±5 nm, *I*=14 μW cm^−2^), green (G; *λ*=545±5 nm, *I*=170 μW cm^−2^), or blue (B; *λ*=470±5 nm, *I*=200 μW cm^−2^) light. Mean values ± SEM for three to twelve independent experiments each performed in triplicate are given. d, e) Phosphorylation of Erk and mFGFR1‐MxCBD or mFGFR1‐TtCBD in response to green light (0–30 min and after recovery for 60 min in the dark; *λ*=545±5 nm, *I*=170 μW cm^−2^).

Next, we quantified the capability of mFGFR1‐MxCBD and mFGFR1‐TtCBD to regulate the MAPK/ERK signaling pathway using a luciferase reporter gene assay (Figure 2 c). For both fusion receptors, we observed induction in the dark that was comparable to that of constitutively dimerized receptor (mFGFR1‐IgG; see the Experimental Section in the Supporting Information). Induction required AdoCbl supplementation, in line with observations that the cofactor is required for domain interaction.8c Upon green‐light illumination, we observed decreased pathway activity comparable with that in unsupplemented or untransfected cells, suggesting light‐induced dissociation of receptor complexes. While the two fusion receptors generally behaved similar, we noticed that blue LED light reduced the activity of the TtCBD fusion receptor, but not that of mFGFR1‐MxCBD, to a similar extent as green light. Control experiments showed that receptor dimerization is required for signaling in the dark (determined using a charge inversion substitution (R195E); see the Experimental Section for details). Furthermore, through illumination at different light intensities, the signaling activity can be tuned to intermediate levels (Figure S5). Collectively, these results demonstrate that green light effectively inhibits the activity of mFGFR1‐CBD receptors and that AdoCbl is required for complex formation in the dark.

We also explored the temporal precision of light‐induced receptor and downstream pathway inactivation (Figure 2 d, e). We confirmed by immunoblotting that the receptors and the downstream signaling protein Erk are phosphorylated in the dark in the presence of AdoCbl, with the phosphorylation decreasing to baseline levels within 5 min of green‐light illumination for Erk and 30 min for mFGFR1. These results also indicate that dissociated receptors remain phosphorylated without downstream signal propagation. We also investigated the phosphorylation recovery upon cessation of illumination. When placed in the dark for 60 min after 30 min illumination, the mFGFR1‐MxCBD phosphorylation recovered to 86 % of the original value, whereas no recovery was observed for mFGFR1‐TtCBD (Figure 2 d, e and Figure S6 a, b). Erk phosphorylation also showed no recovery at this time point, possibly reflecting the complex behavior of the signaling pathway.^14^ We investigated the molecular mechanism underlying this difference, which we hypothesized to reflect the ability of CBDs for cofactor exchange. For TtCBD, it was shown that the corrin ring of AdoCbl forms a stable adduct with a histidine residue (H132; in full‐length CarH), preventing the release of bleached cofactor from its binding pocket.8f Indeed, substitution of this residue (H497A) led to phosphorylation recovery (Figure S7; substitution of the corresponding glutamate residue in mFGFR1‐MxCBD with a histidine (E499H) diminished activity, preventing detailed analysis). Thus CBDs can be used for reversible or irreversible optical control of protein complex dissociation.

To assess the potential of CBDs for optogenetics in vertebrates, we chose to employ mFGFR1‐MxCBD to manipulate zebrafish (*Danio rerio*) embryogenesis as FGF is an important signaling axis during vertebrate development.^15^ We first investigated whether mFGFR1 is functional in zebrafish. Transient overexpression of the constitutively active mFGFR1‐IgG caused developmental malformations such as caudalization of the brain and the formation of a secondary axis, similar to a previously described constitutively active zebrafish FGFR115a (Figure 3 a, b). We next confirmed that injection of AdoCbl and mFGFR1‐MxCBD alone did not negatively impact animal development (Figure 3 c, d). To demonstrate light control over mFGFR1‐MxCBD signaling, we raised animals injected with the receptor and AdoCbl either in the dark or under green‐light irradiation (*λ*=545±5 nm, *I*=180 μW cm^−2^). In the dark, they showed malformations comparable to those of animals injected with mFGFR1‐IgG, suggesting a similar constitutively active mode of action. On the other hand, embryos treated with green light did not show abnormalities, demonstrating that mFGFR1‐MxCBD signaling can be turned off with green light in vivo (Figure 3 e, f). These results indicate that MxCBD is functional in a vertebrate model organism and that mFGFR1‐MxCBD can be used for temporal control to investigate consequences of aberrant FGF signaling during periods of development.

**Figure 3:**
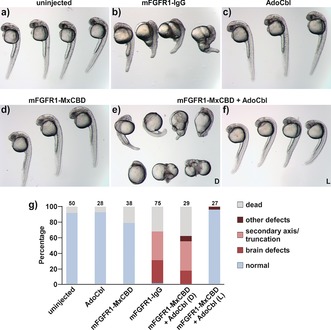
Embryos a) not injected (n.i.) or injected at the one‐cell stage with b) constitutively active mFGFR1‐IgG (13 pg plasmid), c) AdoCbl (50 fmol), d) mFGFR1‐MxCBD (13 pg plasmid), e) mFGFR1‐MxCBD (13 pg plasmid) and AdoCbl (25 fmol) raised in the dark (D), and f) mFGFR1‐MxCBD (13 pg plasmid) and AdoCbl (25 fmol) raised under green light irradiation (L; 545±5 nm, *I*=180 μW cm^−2^ from 1 to 24 hpf). The images were recorded after 24 (a, c, d) and 30 hpf (b, e, f). g) Quantification of phenotypes (numbers denote the number of embryos).

Optogenetic methods that induce the formation of protein complexes in response to blue and red light have matured into important research tools. Herein, we have shown that CBDs enable the dissociation of protein complexes upon green‐light irradiation. The activation of mFGFR1‐CBDs in human cells and zebrafish embryos in the dark resulted in constitutively active signaling and abnormal phenotypes, which could be prevented with green light. The generation of such signals with conventional optogenetic methods would require constant illumination with a risk of bleaching, phototoxicity, and the potential necessity to track the animals. However, we have now shown that green light can be used to inhibit receptor activity either reversibly or irreversibly and with potential for multichromatic experiments. CBDs critically expand the repertoire of photoreceptor domains in optogenetics, and will enable new approaches for cell‐based and animal studies.

## Conflict of interest

The authors declare no conflict of interest.

## Supporting information

As a service to our authors and readers, this journal provides supporting information supplied by the authors. Such materials are peer reviewed and may be re‐organized for online delivery, but are not copy‐edited or typeset. Technical support issues arising from supporting information (other than missing files) should be addressed to the authors.

Supplementary
