# Supplementary material for: Green‐Light‐Induced Inactivation of Receptor Signaling Using Cobalamin‐Binding Domains
Source: Angew Chem Int Ed Engl. 2017 Mar 20;56(16):4608–11. doi: 10.1002/anie.201611998 (PMC5396336; doi:10.1002/anie.201611998)
Supplement: Supplementary file 1 — Supplementary [file ANIE-56-4608-s001.pdf]

Angewandte Chemie
Eine Zeitschrift der Gesellschaft Deutscher Chemiker

Supporting Information

Green-Light-Induced Inactivation of Receptor Signaling Using Cobalamin-Binding Domains

*Stephanie Kainrath, Manuela Stadler, Eva Reichhart, Martin Distel, and Harald Janovjak**

anie_201611998_sm_miscellaneous_information.pdf

Experimental Section

Chemicals

Unless stated otherwise, all chemicals were obtained from Sigma-Aldrich (Vienna, Austria).

CarH CBDs

CBDs of CarH of *M. xanthus* (MxCBD; residues 94 to 299 of NCBI GenBank sequence CAA79965.2) and *T. thermophilus* (TtCBD; residues 80 to 285 of NCBI GenBank sequence WP_038030370.1) were obtained as synthetic gene fragments with mammalin optimization (*H. sapiens*) following the recommendation of the manufacturer (Integrated DNA Technologies, Leuven, Belgium) (Table S1). CBDs were amplified using PCR (oligonucleotides 1-4, Table S2) with overhanging recognition sequences for AgeI and XmaI restriction enzymes.

mFGFR1 fusion proteins

CBDs were inserted into a previously described mFGFR1 expression plasmid in pcDNA3.1(-)[1] using restriction enzymes and ligation. For a positive control construct, the Fc portion of human IgG1 (IgG; residues 230 to 461 of NCBI GenBank sequence KU951249.1 with an additional Cys to Ser substitution) was amplified using PCR (oligonucleotides 5 and 6, Table S2) from human cDNA and inserted into the same construct. The construct contains an N-terminal myristoylation (MYR) membrane anchor and a C-terminal hemagglutinin (HA)-epitope. The CMV promoter of the plasmid was truncated as previously described[2] using inverse PCR (oligonucleotides 7 and 8, Table S2) followed by blunt end ligation to allow more precise adjustment of expression levels in transient transfection experiments. Point substitution R195E in mFGFR1-MxCBD and mFGFR1-TtCBD, H497A in mFGFR1-TtCBD, E499H in mFGFR1-MxCBD (numbered relative to the start codon of the fusion receptors) were introduced using site-directed mutagenesis PCR (oligonucleotides 9-14, Table S2). The charge inversion substitution (R577E in full length mFGFR1; R195E in fusion receptors) prevents formation of a functionally essential, asymmetric kinase domain dimer in FGFR1[3] and was used as a probe for dimer formation during receptor signaling. All constructs were verified by DNA sequencing. Protein sequences are summarized in Table S3.

Cell culture

HEK293 cells were maintained in DMEM supplemented with 10 % FBS, 100 U/ml penicillin and 0.1 mg/ml streptomycin. For luciferase assays, medium was supplemented with AdoCbl or

CNCbl (10 µM final concentration; 10 mM stock concentration in water adjusted to pH 5.5 with HCl[4] and stored at -20°C in small aliquots in the dark). Cells were kept in supplemented medium for at least two passages prior to experiments, and cofactor supplemented medium and cells were kept in the dark to avoid cofactor bleaching.

Incubators for illumination

For illumination of cells, desktop temperature incubators (PT2499; ExoTerra/HAGEN, Holm, Germany or Nordfrost #49340, Schortens, Germany) were equipped with 300 RGB light-emitting diodes. Light intensity was controlled with a dimmer and measured with a power meter (PM120VA, Thorlabs, Dachau, Germany or LP1, Sanwa, Sanwa Electric Instrument, Tokyo, Japan). Intensities were 200 µW cm^{-2} for blue light ($\lambda \approx 470 \pm 5$ nm), 170 µW cm^{-2} for green light ($\lambda \approx 530 \pm 5$ nm) and 14 µW cm^{-2} for red light ($\lambda \approx 630 \pm 5$ nm) for experiments in cells, and 180 µW cm^{-2} for green light ($\lambda \approx 530 \pm 5$ nm) for experiments in zebrafish.

MAPK/ERK pathway activation (luminescence)

Activation of the MAPK/ERK pathway was assessed with the PathDetect Elk1 *trans*-Reporting System (Agilent, Vienna, Austria) containing firefly luciferase and an expression plasmid encoding *Renilla* luciferase as internal standard. Experiments were conducted in 96-well clear bottom plates coated with poly-L-ornithine. 5 x 10^4 HEK293 cells per well from untreated, AdoCbl, or CNCbl supplemented cultures were transfected with 245 ng DNA and 1000 ng polyethylenimine (PEI; Polysciences, Hirschberg an der Bergstrasse, Germany) per well (200 ng *trans*-activator, 10 ng *trans*-reporter, 10 ng RL standard, and 25 ng receptor fusion protein) in CO_2-independent medium (Gibco/Life Technologies, Vienna, Austria; supplemented with 5 % FBS, 2 mM L-glutamine, 100 U/ml penicillin, and 0.1 mg/ml streptomycin). Six h after transfection, medium was changed to CO_2-independent reduced serum starve medium (0.5 % FBS), and cells were incubated for another 14 h under the respective light/dark conditions. Luciferase expression was assessed with a homemade dual-luciferase assay reagent.[5] Firefly and *Renilla* luminescence were measured separately in a microplate reader (Synergy H1, BioTek, Winooski, VT) and the two signals were divided to yield luminescence ratio (LR). For light intensity dependence, cells were incubated with illumination through one, two, or three layers of a neutral density filter foil (transmission for one layer was 0.78) for 6 h after medium change before luciferase quantification.

MAPK/ERK pathway activation (immunoblotting)

1 x 10^6 HEK293 cells were transfected with 2.5 µg receptor in 35 mm dishes or 6 well plates coated with poly-L-ornithine. 6 h after transfection, medium was changed to CO_2-independent reduced serum starve medium (untreated, AdoCbl, or CNCbl supplemented). After 20 h in starve medium, cells were illuminated for different time intervals with LEDs. Following treatment, cells were washed with ice-cold PBS and lysed on ice in 250 µl lysis buffer (150 mM NaCl, 1 % TritonX-100, 0.1 % SDS, 0.5 % sodium deoxycholate, 50 mM Tris, complete protease inhibitor (Roche, Vienna, Austria), pH 8.0) per dish. Lysates were shaken for 30 min at 4°C and centrifuged for 20 min at 12000 rpm at 4°C. 30 µl lysate per lane were separated by SDS-PAGE and electro-blotted onto PVDF membranes. Blots were incubated with primary antibodies (HA #12158-67001, dilution 1:500, Roche; Phospho-FGFR (Tyr653/654) #3471, dilution 1:330, Cell Signaling Technology, Leiden, Netherlands or Phospho-FGFR (Tyr653/654) #PA5-12594, dilution 1:500, ThermoFischer Scientific, Vienna, Austria; pERK1/2 #9101, dilution 1:1000, Cell Signaling Technology; ERK 2 (K-23) sc-153, dilution 1:1000, Santa Cruz Biotechnology, Dallas, Texas, US) in blocking solution (5 % milk powder in TBST) overnight at 4°C. Secondary antibody (goat anti-rabbit IgG(H+L)-HRP conjugate, goat anti-rat IgG(H/L)-HRP, dilution 1:10000, Biorad, Vienna, Austria) was applied for 2 h at 20°C and blots were developed with Clarity™ Western ECL Substrate (Biorad).

Immunoblot quantification

Phosphorylated FGFR1 and total receptor levels for three to four biological replicates were quantified using Image Studio Lite software (LI-COR Biosciences, Bad Homburg, Germany). The ratio of phosphorylated to total receptor were normalized to the dark condition (0 min illumination). The phosphorylated mFGFR1-MxCBD-E499H could not be quantified due to lower protein expression and faint bands.

Fluorescent fusion proteins, expression, and viability testing

CBDs were inserted into a previously described expression plasmid in pcDNA3.1(-)[1] that contains the fluorescent protein mVenus (mV)[6] followed by a glycine- and serine-rich linker and a BspEI restriction site. A previously described mV-FKBP fusion protein was used as positive control[1]. 5 x 10^4 HEK293 cells were transfected with 25 ng expression plasmid. Expression was assessed using mVenus fluorescence in the microplate reader 30 h after transfection. For viability testing, cells were incubated for 2 h with thiazolyl blue tetrazolium bromide (0.5 mg/ml) followed by lysis with 70 µl acidic isopropanol (0.1 N HCl). Absorbance measurements were

taken at 570 nm with 620 nm reference in the microplate reader. Fluorescence microscopy images were recorded on a digital microscope (EVOS FL, Peqlab, Erlangen, Germany) 30 h after transfection.

Animals and analysis of development

mFGFR1-MxCBD and mFGFR1-IgG were subcloned into the pCS2+ expression vector using PCR and the EcoRI and XbaI restriction enzymes. Zebrafish (*Danio rerio*) were maintained at standard rearing conditions[7] and according to the guidelines of the local authorities under licenses GZ:565304/2014/6 and GZ:534619/2014/4. Zebrafish embryos were injected with 1 nl of 13 ng/µl mFGFR1-IgG or mFGFR1-MxCBD with or without 25 µM AdoCbl at the one-cell stage using injection capillaries (glass capillaries pulled with a needle puller; P-97, Sutter Instruments, Novato, CA) mounted onto a micromanipulator (World Precision Instruments Inc., Berlin, Germany) and connected to a microinjector (FemtoJet 4i, Eppendorf, Hamburg, Germany). Injections of AdoCbl were performed using a filter passing red light (106-Primary Red Lee Colour Filter; Thomann, Burgebrach, Germany) on the injection stage and dimmed light. After injection, zebrafish were kept in petri dishes at 28°C. Green illumination was performed using LEDs (see above). To keep zebrafish embryos in the dark, petri dishes were wrapped in aluminum foil. Images of zebrafish embryos were recorded using a stereomicroscope (M125 with LAS software, Leica Microsystems, Wetzlar, Germany). Embryos were divided in four different groups according to their phenotype and counted.

Supporting Figures

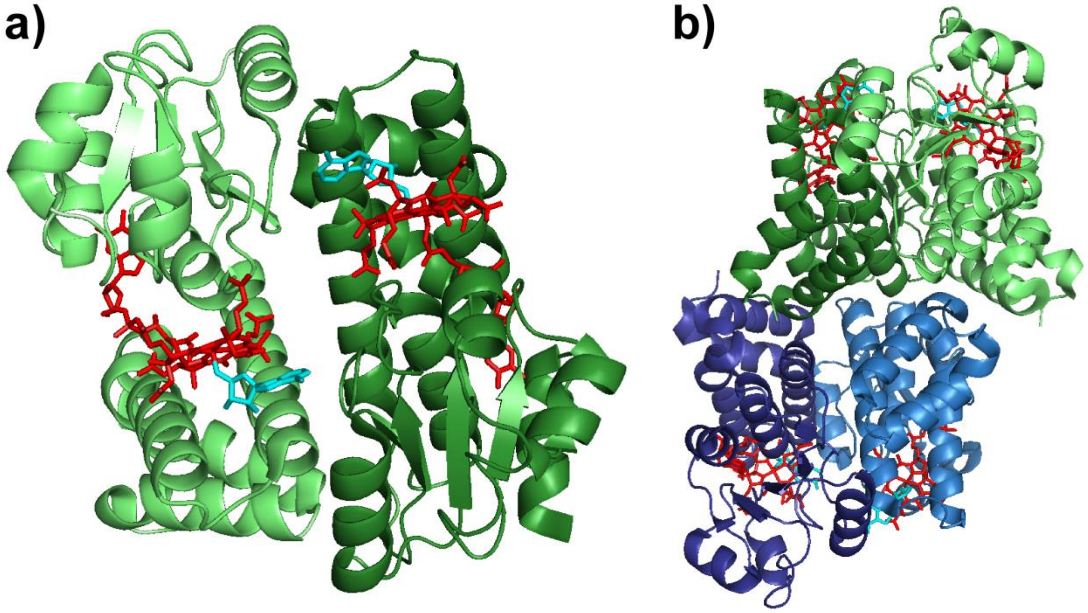

Figure S1: Assembly of TtCBD[8]. a) Head-to-tail dimer with bound AdoCbl (corrin ring and 5'-hydroxyadenosyl group are colored in red and cyan, resp.). b) Tetramer of two head-to-tail dimers.

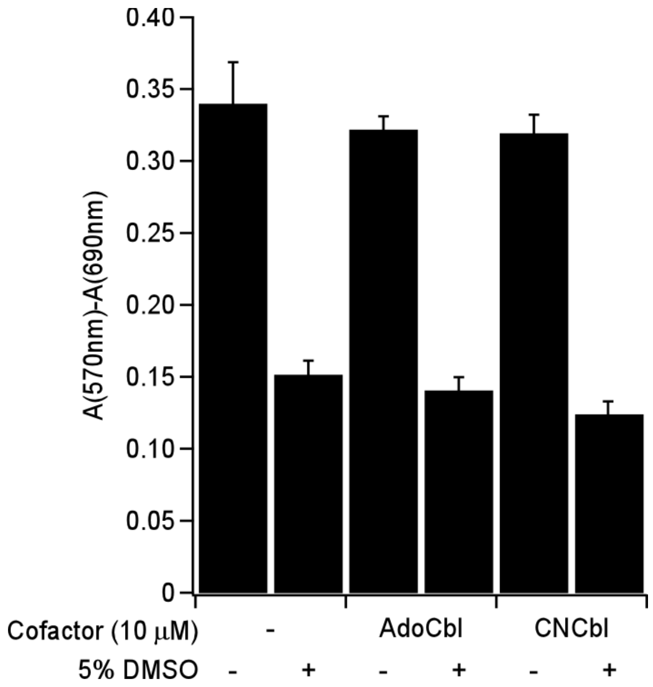

Figure S2: Viability (metabolic activity) of HEK293 cells treated with AdoCbl or CNCbl for 24 h. DMSO was applied as a positive control with reduced viability. Shown are mean values ± SEM for three independent experiments each performed in triplicate.

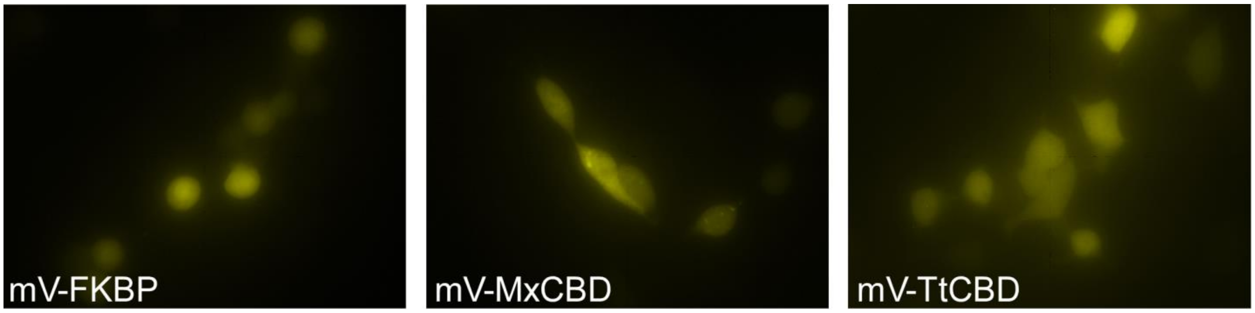

Figure S3: Representative fluorescence microscopy images for mV-FKPB, mV-MxCBD, and mV-TtCBD.

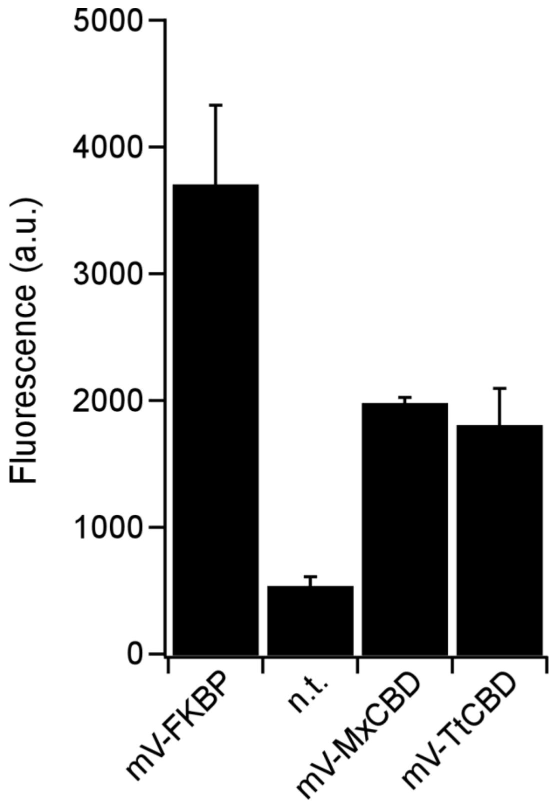

Figure S4: Fluorescence intensity recorded on HEK293 cells expressing mV-FKPB, mV-MxCBD, or mV-TtCBD in medium supplemented with AdoCbl (10 μM, 24 h). Experiments were performed as for data shown in Figure 1 d. Not transfected cells (n.t.) are the negative control. Shown are mean values ± SEM for three independent experiments each performed in triplicate.

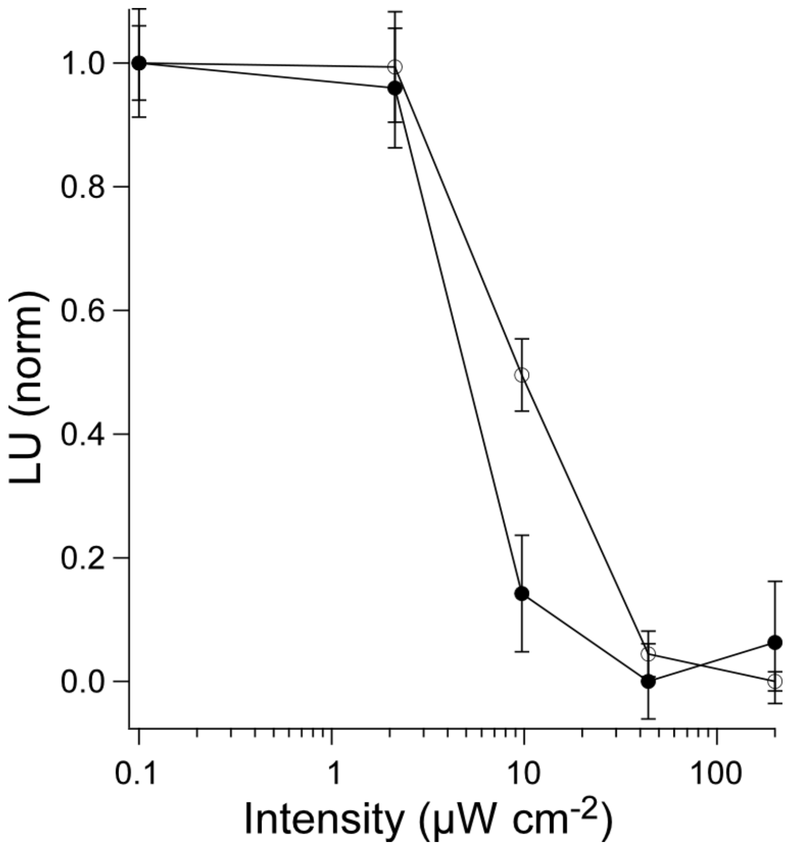

Figure S5: Light intensity dependence of green light-induced inactivation of FGFR1 signaling. Experiments were performed as for data shown in Figure 2 c and data were normalized to highest and lowest responses. Closed symbols: mFGFR1-MxCBD. Open symbols: mFGFR1-TtCBD. Shown are mean values ± SEM for three independent experiments each performed in triplicate.

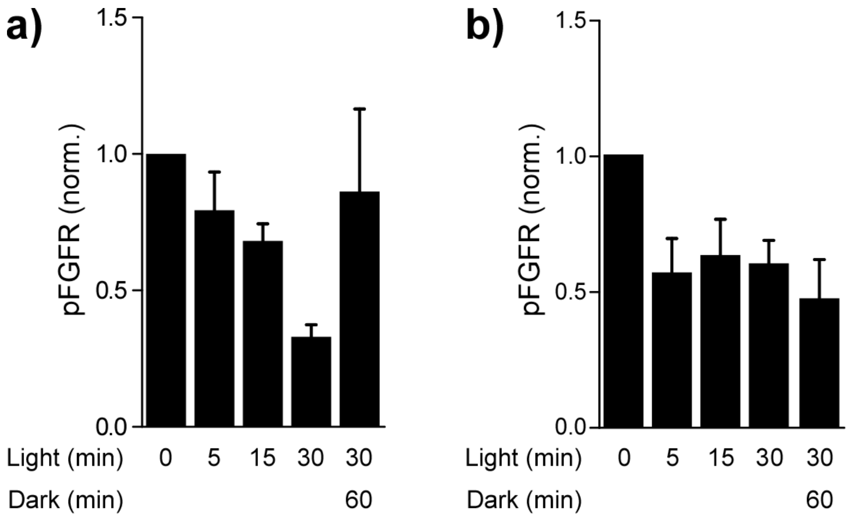

Figure S6: Receptor phosphorylation in HEK293 cells transfected with mFGFR1-MxCBD (a) and mFGFR1-TtCBD (b). Shown are mean values ± SEM for three to four independent experiments.

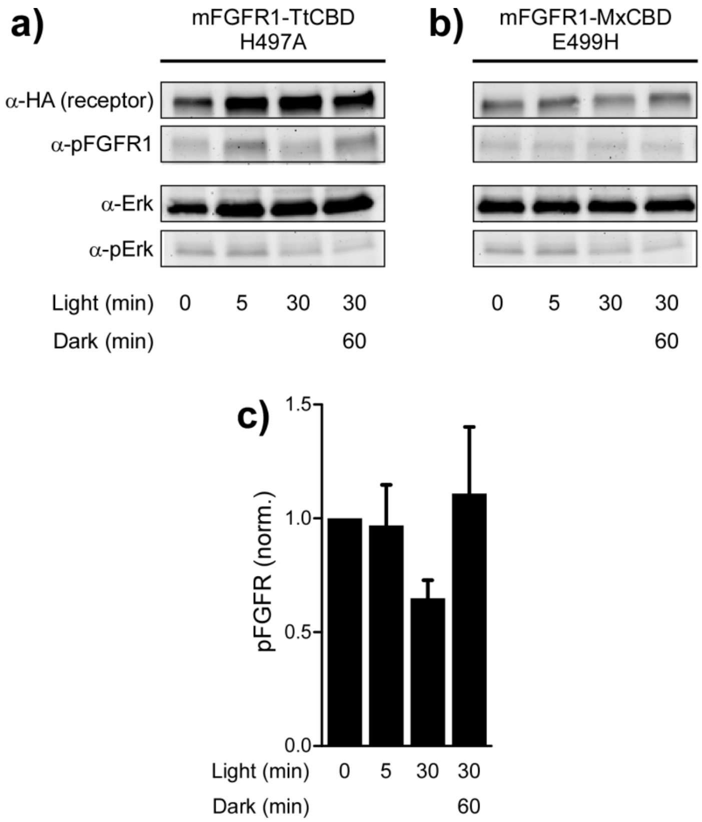

Figure S7: (a and b) Phosphorylation of Erk and mFGFR1 in HEK293 cells transfected with mFGFR1-TtCBD with H497A substitution (a) or mFGFR1-MxCBD with E499H substitution (b) in reponse to green light (5 or 30 min) and after dark recovery (60 min). (c) Quantification of data shown in a) (mean values ± SEM for three independent experiments; phosphorylation of mFGFR1-MxCBD-E499H could not be quantified due to lower protein expression and faint bands).

Supporting Tables

Table S1: Codon-optimized CBD sequences.

Name	Sequence
MxCBD	CCACACGCAGAGACTTGGAGGGAATCTATGCTTGCCGCCACACAAGCCTAC<br>GACCAGCCTAGAGTATCAGATGTACTGGATGAGGTCCTTGCGGCTCTGCCC<br>CCTCTGAAGGCCTTCGATGAAGTGCTGGCCCCTTTGCTGTGCGATGTCGGA<br>GAGCGGTGGGAGAGCGGAACCCTGACAGTTGCGCAGGAACATCTGGTCTC<br>ACAGATGGTGCGCGCCCGGCTGGTGAGTCTGCTGCACGCGGCACCATTGG<br>GACGCCACAGACATGGCGTTCTCGCCTGTTTCCCAGAGGAGGAGCATGAGA<br>TGGGCTTGCTTGGTGCCGCCTTGAGACTCCGCCATCTCGGCGTTAGAGTAA<br>CCCTGCTCGGCCAGCGAGTGCCAGCCGAGGACCTCGGGCGAGCAGTGTTG<br>GCCCTGCGCCCGGACTTCGTGGGCCTGTCAACAGTTGCAAGCAGGAGCGC<br>AGAGGACTTCGAGGATACCTTGACCCGACTCCGCCAGGCCCTGCCAAGGG<br>GCCTCCCTGTATGGGTGGGCGGGGCAGCCGCAAGGTCTCATCAGGCCGTG<br>TGCGAGCGCCTGGCAGTCCATGTTTTTCAGGGCGAAGAAGATTGGGATAGA<br>CTTGCCGGAACA
TtCBD	CCCGAGGACCTCGGCACCGGACTCCTCGAAGCACTCTTGAGAGGAGATTTG<br>GCCGGCGCCGAGGCTCTCTTTCGACGGGGGCTTAGGTTTTGGGGACCCGA<br>GGGTATTCTGGAGCACCTGCTCCTGCCTGTGCTTCGGGAGGTGGGAGAAGC<br>CTGGCATCGCGGCGAGATCGGTGTGGCCGAGGAGCATCTGGCATCCACATT<br>TCTGCGCGCGAGACTGCAGGAGCTGCTCGACCTCGCCGGGTTCCCACCTG<br>GCCCCCCCGTGCTGGTAACCACGCCACCAGGGGAGAGGCACGAGATCGGC<br>GCAATGTTGGCTGCCTATCACCTGCGAAGGAAGGGCGTGCCAGCGCTGTAC<br>TTGGGACCAGACACCCCCCTCCCCGATCTCAGAGCACTTGCGAGACGGCTC<br>GGAGCCGGAGCGGTGGTTCTGTCAGCTTTGCTTTCCGAGCCTCTCAGGGCG<br>TTGCCAGACGGCGCACTGAAAGACTTGGCACCTCGGGTGTTCCTGGGAGG<br>GCAAGGAGCCGGCCCTGAAGAGGCCCGACGGCTCGGGGCCGAGTACATG<br>GAAGATCTGAAGGGATTGGCCGAAGCACTGTGGCTTCCAAGAGGACCAGAG<br>AAAGAAGCAATC

Table S2: Oligonucleotides utilized for PCR. Restriction sites are underlined.

	Name	Sequence
1	MxCBD_F	GATCATACCGGTCCACACGCA
2	MxCBD_R	GATCATCCCGGGTGTTCCGGCA
3	TtCBD_F	GATCATACCGGTCCCGAGGACCT
4	TtCBD_R	GATCATCCCGGGGATTGCTTCTTT
5	IgG_F	GACGACCGGTGAGCCCAAATCTTCTGACAAAACTCAC
6	IgG_R	GATCACCGGTTTTACCCGGAGACAGGGAGAG
7	truncated CMV_pcDNA3.1(-)_F	TGGGAGGTCTATATAAGCAGAGC
8	truncated CMV_pcDNA3.1(-)_R	GGCGGGCCATTTACCGTAAG
9	mFGFR1_R195E_F	TACAGGCCCGGGAGCCTCCTGGGCTGGAGTACTGCTATAA
10	mFGFR1_R195E_R	TTATAGCAGTACTCCAGCCCAGGAGGCTCCCGGGCCTGTA
11	mFGFR1-TtCBD_H497A_F	GAGAAGCCTGGGCTCGCGGCGAGAT
12	mFGFR1-TtCBD_H497A_R	ATCTCGCCGCGAGCCCAGGCTTCTC
13	mFGFR1-MxCBD_E499H_F	GAGAGCGGTGGCACAGCGGAACCCT
14	mFGFR1-MxCBD_E499H_R	AGGGTTCCGCTGTGCCACCGCTCTC

Table S3: Protein sequences of full length proteins (NCBI Reference Sequence given in brackets), CBDs, receptors, and fluorescent fusion proteins.

Name	Sequence
MxCarH<br><br>(CAA79965.2)	MAERTYRINIAAELAGVRVELIRAWERRYGVLTPRRTPAGYRAYTDRD<br>VAVLKQLKRLTDEGVAISEAAKLLPQLMEGLEAEVAGRGASQDARPH<br>AETWRESMLAATQAYDQPRVSDVLDEVLAALPPLKAFDEVLAPLLCD<br>VGERWESGTLTVAQEHLVSQMVRARLVSLLHAAPLGRHRHGVLACF<br>PEEEHEMGLLGAALRLRHLGVRVTLLGQRVPAEDLGRAVLALRPDFV<br>GLSTVASRSAEDFEDTLTRLRQALPRGLPVWVGGAAARSHQAVCER<br>LAVHVFQGEEDWDRLAGT
MxCBD	HAETWRESMLAATQAYDQPRVSDVLDEVLAALPPLKAFDEVLAPLLC<br>DVGERWESGTLTVAQEHLVSQMVRARLVSLLHAAPLGRHRHGVLAC<br>FPEEEHEMGLLGAALRLRHLGVRVTLLGQRVPAEDLGRAVLALRPDF<br>VGLSTVASRSAEDFEDTLTRLRQALPRGLPVWVGGAAARSHQAVCE<br>RLAVHVFQGEEDWDRLAGT
TtCarH<br><br>(WP_038030370.1)	MTSSGVYTIAEVEAMTGLSAEALRQWERRYGFPKPRRTPGGHRLYS<br>AEDVEALKTIKRWLEEGATPKAAIRRYLAQGVRPEDLGTGLLEALLRG<br>DLAGAEALFRRGLRFWGPEGILEHLLLPVLREVGEAWHRGEIGVAEE<br>HLASTFLRARLQELLDLAGFPPGPPVLVTTPPGERHEIGAMLAAYHLR<br>RKGVPALYLGPDTPLPDLRALARRLGAGAVVLSALLSEPLRALPDGAL<br>KDLAPRVFLGGQGAGPEEARRLGAEYMEDLKGLAEALWLPRGPEKE<br>AI
TtCBD	PEDLGTGLLEALLRGDLAGAEALFRRGLRFWGPEGILEHLLLPVLREV<br>GEAWHRGEIGVAEEHLASTFLRARLQELLDLAGFPPGPPVLVTTPPG<br>ERHEIGAMLAAYHLRRKGVPALYLGPDTPLPDLRALARRLGAGAVVLS<br>ALLSEPLRALPDGALKDLAPRVFLGGQGAGPEEARRLGAEYMEDLKG<br>LAEALWLPRGPEKEAI
mFGFR1-MxCBD	MGSSKSKPKDPSQRLDMKSGTKKSDFHSQMAVHKLAKSIPLRRQVT<br>VSADSSASMNSGVLLVRPSRLSSSGTPMLAGVSEYELPEDPRWELP<br>RDRLVLGKPLGEGCFGQVVLAEAIGLDKDKPNRVTKVAVKMLKSDAT<br>EKDLSDLISEMEMMKMIGKHKNIINLLGACTQDGPLYVIVEYASKGNLR<br>EYLQARRPPGLEYCYNPSHNPEEQLSSKDLVSCAYQVARGMEYLAS<br>KKCIHRDLAARNVLVTEDNVMKIADFGLARDIHHIDYYKKTTNGRLPVK

	WMAPEALFDRIYTHQSDVWSFGVLLWEIFTLGGSPYPGVPVEELFKL LKEGHRMDKPSNCTNELYMMMRDCWHAVPSQRPTFKQLVEDLDRIV ALTSNQEYLDLSIPLDQYSPSFPDTRSSTCSSGEDSVFSHEPLPEEPC LPRHPTQLANSGLKRRVETGPHAETWRESMLAATQAYDQPRVSDVL DEVLAALPPLKAFDEVLAPLLCDVGERWESGTLTVAQEHLVSQMVRA RLVSLLHAAPLGRHRHGVLACFPEEEHEMGLLGAALRLRHLGVRVTL LGQRVPAEDLGRAVLALRPDFVGLSTVASRSAEDFEDTLTRLRQALP RGLPVWVGGAAARSHQAVCERLAVHVFQGEEDWDRLAGTPGGSGV DYPYDVPDYALD
mFGFR1-TtCBD	MGSSKSKPKDPSQRLDMKSGTKKSDFHSQMAVHKLAKSIPLRRQVT VSADSSASMNSGVLLVRPSRLSSSGTPMLAGVSEYELPEDPRWELP RDRLVLGKPLGEGCFGQVVLAEAIGLDKDKPNRVTKVAVKMLKSDAT EKDLSDLISEMEMMKMIGKHKNIINLLGACTQDGPLYVIVEYASKGNLR EYLQARRPPGLEYCYNPSHNPEEQLSSKDLVSCAYQVARGMEYLAS KKCIHRDLAARNVLVTEDNVMKIADFGLARDIHHIDYYKKTTNGRLPVK WMAPEALFDRIYTHQSDVWSFGVLLWEIFTLGGSPYPGVPVEELFKL LKEGHRMDKPSNCTNELYMMMRDCWHAVPSQRPTFKQLVEDLDRIV ALTSNQEYLDLSIPLDQYSPSFPDTRSSTCSSGEDSVFSHEPLPEEPC LPRHPTQLANSGLKRRVETGPEDLGTGLLEALLRGDLAGAEALFRRG LRFWGPEGILEHLLLPVLREVGEAWHRGEIGVAEEHLASTFLRARLQE LLDLAGFPPGPPVLVTTPPGERHEIGAMLAAYHLRRKGVPALYLGPDT PLPDLRALARRLGAGAVVLSALLSEPLRALPDGALKDLAPRVFLGGQG AGPEEARRLGAEYMEDLKGLAEALWLPRGPEKEAIPGGSGVDYPYD VPDYALD
mFGFR1-IgG	MGSSKSKPKDPSQRLDMKSGTKKSDFHSQMAVHKLAKSIPLRRQVT VSADSSASMNSGVLLVRPSRLSSSGTPMLAGVSEYELPEDPRWELP RDRLVLGKPLGEGCFGQVVLAEAIGLDKDKPNRVTKVAVKMLKSDAT EKDLSDLISEMEMMKMIGKHKNIINLLGACTQDGPLYVIVEYASKGNLR EYLQARRPPGLEYCYNPSHNPEEQLSSKDLVSCAYQVARGMEYLAS KKCIHRDLAARNVLVTEDNVMKIADFGLARDIHHIDYYKKTTNGRLPVK WMAPEALFDRIYTHQSDVWSFGVLLWEIFTLGGSPYPGVPVEELFKL LKEGHRMDKPSNCTNELYMMMRDCWHAVPSQRPTFKQLVEDLDRIV ALTSNQEYLDLSIPLDQYSPSFPDTRSSTCSSGEDSVFSHEPLPEEPC LPRHPTQLANSGLKRRVETGEPKSSDKTHTCPPCPAPELLGGPSVFL

	FPPKPKDTLMISRTPEVTCVVVDVSHEDPEVKFNWYVDGVEVHNAKT<br>KPREEQYNSTYRVVSVLTVLHQDWLNGKEYKCKVSNKALPAPIEKTIS<br>KAKGQPREPQVYTLPPSRDELTKNQVSLTCLVKGFYPSDIAVEWESN<br>GQPENNYKTTPPVLDSDGSFFLYSKLTVDKSRWQQGNVFSCSVMHE<br>ALHNHYTQKSLSLSPGKTGGSGVDYPYDVPDYALD
mV-MxCBD	MVSKGEELFTGVVPILVELDGDVNGHKFSVSGEGEGDATYGKLTLKLI<br>CTTGKLPVPWPTLVTTLGYGLQCFARYPDHMKQHDFFKSAMPEGYV<br>QERTIFFKDDGNYKTRAEVKFEGDTLVNRIELKGIDFKEDGNILGHKLE<br>YNYNSHNVYITADKQKNGIKANFKIRHNIEDGGVQLADHYQQNTPIGD<br>GPVLLPDNHYLSYQSKLSKDPNEKRDHMVLLEFVTAAGITLGMDELYK<br>GSSGSSGPHAETWRESMLAATQAYDQPRVSDVLDEVLAALPPLKAF<br>DEVLAPLLCDVGERWESGTLTVAQEHLVSQMVRARLVSLLHAAPLGR<br>HRHGVLACFPEEEHEMGLLGAALRLRHLGVRVTLLGQRVPAEDLGRA<br>VLALRPDFVGLSTVASRSAEDFEDTLTRLRQALPRGLPVWVGGAAAR<br>SHQAVCERLAVHVFQGEEDWDRLAGTPG
mV-TtCBD	MVSKGEELFTGVVPILVELDGDVNGHKFSVSGEGEGDATYGKLTLKLI<br>CTTGKLPVPWPTLVTTLGYGLQCFARYPDHMKQHDFFKSAMPEGYV<br>QERTIFFKDDGNYKTRAEVKFEGDTLVNRIELKGIDFKEDGNILGHKLE<br>YNYNSHNVYITADKQKNGIKANFKIRHNIEDGGVQLADHYQQNTPIGD<br>GPVLLPDNHYLSYQSKLSKDPNEKRDHMVLLEFVTAAGITLGMDELYK<br>GSSGSSGPEDLGTGLLEALLRGDLAGAEALFRRGLRFWGPEGILEHL<br>LLPVLREVGEAWHRGEIGVAEEHLASTFLRARLQELLDLAGFPPGPPV<br>LVTTPPGERHEIGAMLAAYHLRRKGVPALYLGPDTPLPDLRALARRLG<br>AGAVVLSALLSEPLRALPDGALKDLAPRVFLGGQGAGPEEARRLGAE<br>YMEDLKGLAEALWLPRGPEKEAIPG

References

[1] M. Grusch, K. Schelch, R. Riedler, E. Reichhart, C. Differ, W. Berger, A. Ingles-Prieto, H. Janovjak, *EMBO J.* **2014**, *33*, 1713-1726.

[2] N. Watanabe, T. J. Mitchison, *Science* **2002**, *295*, 1083-1086.

[3] J. H. Bae, T. J. Boggon, F. Tome, V. Mandiyan, I. Lax, J. Schlessinger, *Proc. Natl. Acad. Sci. USA* **2010**, *107*, 2866-2871.

[4] Z. Schneider, A. Stroiński, *Comprehensive B12 : Chemistry, biochemistry, nutrition, ecology, medicine*, De Gruyter, Berlin; New York, **1987**.

[5] J. M. Baker, F. M. Boyce, *Journal of visualized experiments : JoVE* **2014**.

[6] T. Nagai, K. Ibata, E. S. Park, M. Kubota, K. Mikoshiba, A. Miyawaki, *Nature Biotech.* **2002**, *20*, 87-90.

[7] a) C. B. Kimmel, W. W. Ballard, S. R. Kimmel, B. Ullmann, T. F. Schilling, *Developmental dynamics: An official publication of the American Association of Anatomists* **1995**, *203*, 253-310; b) M. Westerfield, *The zebrafish book. A guide for the laboratory use of zebrafish (Danio rerio)*, 4th ed., Univ. of Oregon Press, Eugene, **2000**.

[8] M. Jost, J. Fernandez-Zapata, M. C. Polanco, J. M. Ortiz-Guerrero, P. Y. Chen, G. Kang, S. Padmanabhan, M. Elias-Arnanz, C. L. Drennan, *Nature* **2015**, *526*, 536-541.
